# Bibliography

**Published:** 1909-02-15

**Authors:** 


### BIBLIOGRAPHY.

Practical Dentistry.—This is the title given a complication of formula and extracts on technical procedure as published in our current periodic literature during the past decade, and which has been gathered and printed in book form by Dr. I. N. Broomell, and the publishers of the *Dental Brief*. The selections are certainly comprehensive and abundant. The book is divided into two parts, one for operative procedure and the other for prosthodontia. There are necessarily many formulas and procedures listed that could be criticised, and there are also quite a number of unintentional errors that we have noticed in a casual reading. It isn't a book that any one would read consecutively, but will make a good reference book for special things. The book could have been made much more valuable had it been completely indexed as well as classified more closely. The book is printed by the L. D. Caulk Co., and can be had through the dental supply houses.

Gray's Anatomy.—The new edition of Gray's Anatomy is before us, and it has grown to a work of over 1,600 pages. One naturally thinks of anatomy as one of the fixed sciences and text books on this subject have no business to get out of date; but when we compare this seventeenth edition with its nearest predecessor, we find that anatomy is one of the live subjects of study and research. This new edition celebrates the semi-centenial of this great work's existence, no other text book on medical science in any branch has held the supreme place this one has occupied for so many years. We notice some eliminations and a great many additions since the last edition. The illustrations of former editions which have always been characteristic, are retained and many new colored plates are added, especially to the surgical regions that have in recent years claimed especial attention, abdominal surgery in particular. The section on the nervous system has been entirely re-written. New physiological facts have made many changes in text matter necessary, and many new illustrations have helped to elucidate the text matter. The new anatomical nomenclature has been recognized to the extent of introducing the new terms in parenthesis. The publishers certainly acted with wisdom in selecting the eminent surgeon Dr. DaCosta and the equally eminent anatomist and artist, Dr. Spitzka to supervise this new work, and they will undoubtedly have the credit of producing the greatest work of its kind. The mechanical features of the work are fully up to the standard of the esteemed house of Lea & Febiger, successor to the old house of Lea Bros. & Co., makers of our best medical books for many years.

